# Effects of Face Mask Mandates on COVID-19 Transmission in 51 Countries: Retrospective Event Study

**DOI:** 10.2196/49307

**Published:** 2024-03-08

**Authors:** Anatol-Fiete Näher, Matthias Schulte-Althoff, Marvin Kopka, Felix Balzer, Francisco Pozo-Martin

**Affiliations:** 1 Digital Global Public Health Hasso Plattner Institute University of Potsdam Potsdam Germany; 2 Institute for Medical Informatics Charité - Universitätsmedizin Berlin Germany; 3 Method Development, Research Infrastructure, and Information Technology Robert Koch Institute Berlin Germany; 4 Department of Information Systems School of Business and Economics Freie Universität Berlin Germany; 5 Department of Psychology and Ergonomics Technische Universität Berlin Berlin Germany; 6 Evidence-based Public Health Unit Center for International Health Protection Robert Koch Institute Berlin Germany

**Keywords:** nonpharmacological interventions, face masks, infectious diseases, acute respiratory infections, COVID-19, real-world evidence

## Abstract

**Background:**

The question of the utility of face masks in preventing acute respiratory infections has received renewed attention during the COVID-19 pandemic. However, given the inconclusive evidence from existing randomized controlled trials, evidence based on real-world data with high external validity is missing.

**Objective:**

To add real-world evidence, this study aims to examine whether mask mandates in 51 countries and mask recommendations in 10 countries increased self-reported face mask use and reduced SARS-CoV-2 reproduction numbers and COVID-19 case growth rates.

**Methods:**

We applied an event study approach to data pooled from four sources: (1) country-level information on self-reported mask use was obtained from the COVID-19 Trends and Impact Survey, (2) data from the Oxford COVID-19 Government Response Tracker provided information on face mask mandates and recommendations and any other nonpharmacological interventions implemented, (3) mobility indicators from Google’s Community Mobility Reports were also included, and (4) SARS-CoV-2 reproduction numbers and COVID-19 case growth rates were retrieved from the Our World in Data—COVID-19 data set.

**Results:**

Mandates increased mask use by 8.81 percentage points (*P*=.006) on average, and SARS-CoV-2 reproduction numbers declined on average by −0.31 units (*P*=.008). Although no significant average effect of mask mandates was observed for growth rates of COVID-19 cases (−0.98 percentage points; *P*=.56), the results indicate incremental effects on days 26 (−1.76 percentage points; *P*=.04), 27 (−1.89 percentage points; *P*=.05), 29 (−1.78 percentage points; *P*=.04), and 30 (−2.14 percentage points; *P*=.02) after mandate implementation. For self-reported face mask use and reproduction numbers, incremental effects are seen 6 and 13 days after mandate implementation. Both incremental effects persist for >30 days. Furthermore, mask recommendations increased self-reported mask use on average (5.84 percentage points; *P*<.001). However, there were no effects of recommendations on SARS-CoV-2 reproduction numbers or COVID-19 case growth rates (−0.06 units; *P*=.70 and −2.45 percentage points; *P*=.59). Single incremental effects on self-reported mask use were observed on days 11 (3.96 percentage points; *P*=.04), 13 (3.77 percentage points; *P*=.04) and 25 to 27 (4.20 percentage points; *P*=.048 and 5.91 percentage points; *P*=.01) after recommendation. Recommendations also affected reproduction numbers on days 0 (−0.07 units; *P*=.03) and 1 (−0.07 units; *P*=.03) and between days 21 (−0.09 units; *P*=.04) and 28 (−0.11 units; *P*=.05) and case growth rates between days 1 and 4 (−1.60 percentage points; *P*=.03 and −2.19 percentage points; *P*=.03) and on day 23 (−2.83 percentage points; *P*=.05) after publication.

**Conclusions:**

Contrary to recommendations, mask mandates can be used as an effective measure to reduce SARS-CoV-2 reproduction numbers. However, mandates alone are not sufficient to reduce growth rates of COVID-19 cases. Our study adds external validity to the existing randomized controlled trials on the effectiveness of face masks to reduce the spread of SARS-CoV-2.

## Introduction

### Background

Viral and bacterial acute respiratory infections (ARIs) are among the leading causes of death worldwide. With a total of 3.8%, ARIs contributed the fourth largest share to the global burden of disease in 2019 [1]. Thus, ARIs pose a significant threat to global health. A measure to prevent ARI, which has also been discussed extensively in the context of the COVID-19 pandemic, is the wearing of face masks. At the heart of this discussion are the following 2 considerations: first, a large proportion of COVID-19 infections are transmitted by asymptomatic individuals [2,3], and second, masks worn by infected individuals are thought to reduce transmission risks. Therefore, in the face of a potentially large number of undetected cases, it has been argued that wearing masks could contribute to a sustained decrease in the spread of COVID-19 [4]. In contrast, a recently updated Cochrane review did not reach a clear conclusion regarding the preventive efficacy of face masks for viral respiratory infections [5]. On the basis of 78 randomized controlled trials (RCTs) conducted during influenza seasons as well as during the H1N1 influenza and COVID-19 pandemics, the authors conclude that masks alone are not sufficient to reduce the spread of respiratory viruses. However, given the results, it should be noted that interpretation is complicated by the fact that adherence was low, and outcome measures varied across a large proportion of RCTs. Furthermore, the observations of Jefferson et al [5] are at odds with clinical trials demonstrating the efficacy of masks in protecting uninfected individuals from COVID-19 [6,7]. Clinical trials have also shown that masks reduce the risk of transmission of influenza or SARS-CoV-2 from previously infected individuals [8].

A key advantage of studies that are based on real-world data is their high external validity. As such, they can significantly complement the evidence from RCTs on the efficacy of face masks in protecting against ARIs. ARIs can be most effectively prevented at the population level when masks are worn by both susceptible and infectious individuals [8,9]. Following this line of reasoning, the World Health Organization issued a recommendation to publicly wear face masks to contain the spread of SARS-CoV-2 [10]. This recommendation has been adopted by countries around the world by implementing face mask mandates in 2020. As some countries and regions did not introduce any face mask policies, 3 studies used this fact to identify the causal effect of mandates on SARS-CoV-2 infections. Lyu and Wehby [11] conducted a natural experiment with real-world data to show that mandates in 15 states of the United States and Washington, District of Columbia, resulted in reductions in COVID-19 growth rates of up to 19%. Another study using real-world data from the Global COVID-19 Trends and Impact Survey (CTIS) found mask mandates in the United States to be related to decreases in daily new cases, daily new deaths, daily new hospital admissions, and increases in population shares wearing masks [12]. These findings are corroborated by Mitze et al [13], who, using a synthetic control design, found a 47% reduction in daily COVID-19 case growth rates attributable to a local mask mandate in Jena, Germany. In addition, 2 other population-level studies found evidence for a direct association between wearing face masks and COVID-19 outcomes. Controlling for time-constant unobserved heterogeneity, Rader et al [14] report decreasing growth rates of COVID-19 cases with increasing state-specific proportions of face mask wearers at the US state level. Leffler et al [15] use a cross-sectional design across 196 countries and observe that longer durations of face mask use are inversely associated with COVID-19 mortality.

### Objectives

To our knowledge, no real-world studies have been conducted to compare the effects of face mask mandates on SARS-CoV-2 infections internationally. Our study provides missing evidence. A prerequisite for mask mandates to be effective is that they encourage populations to wear masks [5,16]. Therefore, we exploited longitudinal variations in outcomes to first evaluate the hypothesis that mask mandates increase mask use in 51 countries worldwide. We then used the same design to test the hypothesis that mask mandates lead to a decrease in SARS-CoV-2 reproduction numbers and growth rates in COVID-19–related cases. In planning future nonpharmacological interventions, it is also important to be able to assess whether mandates are necessary to achieve the desired effect on health outcomes or whether recommendations are sufficient. Thus, the hypothesis that recommendations for face masks would lead to an increase in self-reported face mask use and a reduction in SARS-CoV-2 reproduction numbers and COVID-19 case growth rates was also evaluated. COVID-19 is a specific type of ARI. Except for rhinovirus infections, ARIs are comparable in terms of their transmission routes. Therefore, the results of our study not only provide an indication of the effectiveness of face mask interventions in the context of the COVID-19 pandemic but may also be indicative of how future ARI outbreak scenarios could potentially be addressed.

## Methods

### Measures

We gathered information on the proportions of face mask wearers in 105 countries from the CTIS, which is administered by the Carnegie Mellon University and the University of Maryland in partnership with Meta [17]. The CTIS uses an administrative region-stratified random sample that was drawn daily from Facebook’s active user base. Via Facebook’s news feed, the drawn individuals were invited to participate in the survey. After obtaining informed consent, users were provided with a link to a web-based questionnaire on COVID-19–related attitudes, symptoms, and behaviors. Daily population shares of self-reported face mask users were calculated from the responses of survey participants who stated that they had worn a face mask always or most of the time when in public. Before aggregation, the individual-level observations were reweighted by means of survey weights provided by Meta. Although no information collected by the survey is shared with Meta, Meta itself calculated weights that adjust for nonresponse and sampling frame mismatches with country-specific populations based on age, gender, and administrative regions [18]. Weights were made available to researchers without disclosing any user-related content.

To allow for linear model specifications, we chose 2 country-specific COVID-19 outcome measures: SARS-CoV-2 reproduction numbers and 3-day growth rates of COVID-19–associated cases per 100,000 inhabitants. To obtain information on the progression of reproduction numbers over time, we used the Our World in Data—COVID-19 data set [19]. The daily effective reproduction numbers included in the data set were derived using Kalman filter estimates of weekly case growth rates, as described by Arroyo-Marioli et al [20].

Data on nonpharmacological interventions were obtained from the Oxford COVID-19 Government Response Tracker (OxCGRT) [21]. Between January 1, 2020, and December 31, 2022, the tracker collected the start and end dates of COVID-19–related policy interventions in 186 countries. The OxCGRT data set also includes a measure that captures the scope of face mask–related policies on an ordinal scale from 1 to 4. On this scale, 1 designates official face mask recommendations. Mandates are indicated by values ranging from 2 to 4. Using this scale, we defined the levels of mandates according to the extent to which exemptions from the obligation to wear face masks in public were made. Corresponding to a value of 2, level 1 applies to all mandates that required face masks to be worn in some public places where other people were present or where social distancing was not possible, such as certain shops or public transport. Level 2 is equivalent to a value of 3, which corresponds to mandates that were more restrictive and generally required people to wear face masks in public places where other people were present or where social distancing was not possible. Level-2 mandates only allowed people not to wear masks in places such as less crowded streets or parks. Level 3 corresponds to a value of 4 and means that face masks had to be worn outdoors at all times, regardless of location or the presence of other people. We created dummy variables for each mandate level to indicate whether the mandates at a particular level were enacted at a particular time. The OxCGRT data further include an item that differentiates between policies targeted at subgroups or entire populations. A control variable for country-specific face mask mandates that applied only to subgroups was generated from this item and the indicators for level-1 face mask mandates. The variable takes the value 1 at dates when the corresponding subgroup mandates were active; otherwise, it takes the value 0. This allows for the inclusion of all subgroup mandates. None of the included countries implemented subgroup mandates at a level higher than level 1. Furthermore, we added date-specific indicators of the following additional nonpharmacological interventions: school closures, bans on events and gatherings, international travel restrictions, curfews, and measures to protect older adults. Measures to protect older adults include recommendations or restrictions on the number of visitors and hygiene practices in nursing homes. At a given time, indicators for additional nonpharmacological interventions indicate whether the intervention is recommended or mandatory, as opposed to not recommended or mandatory. Country-level mobility indicators are also included in the analyses. The mobility indicator is calculated by Google from aggregated location data that users have agreed to share through their devices. The metric indicates the daily relative changes in mobility as compared with the baseline period between January 3 and February 6, 2020 [22].

### Observational Sample

For the analyses, we constructed a longitudinal data set by merging daily repeated cross-sections of the CTIS with the OxCGRT, Our World in Data, and Google mobility data sets. Beginning on April 23, 2020, when the first CTIS data became available, the observation period was restricted to October 31, 2020, yielding a total of 192 observation dates in calendar time. In total, 102 countries with national-level policy interventions targeted at general populations are commonly represented in all 3 data sets. Of these, 78.4% (80/102) enacted national mandates on the use of face masks in public. As the recording of the CTIS data commenced only after the interventions, the effects of face mask policies could not be identified for 43% (34/80) of the mandate countries. These countries were excluded from the analysis. Another 4% (3/80) of the mandate countries were excluded because information on SARS-CoV-2 reproduction numbers was not available at the time when mask mandates became effective.

The CTIS data and information on SARS-CoV-2 reproduction numbers and COVID-19 case growth rates were available for 100% (8/8) of the countries that did not enact any face mask policies throughout the observation period. These countries served as the control group. As can be inferred from Table 1 [17,21], this resulted in a total of 51 observed countries worldwide, out of which 84% (43/51) countries implemented level-1 face mask interventions. The countries included were Albania, Azerbaijan, Belgium, Bangladesh, Belarus, Bolivia, Costa Rica, Germany, Denmark, Egypt, Finland, France, Ghana, Greece, Honduras, Croatia, Hungary, Ireland, Jordan, Japan, Kazakhstan, South Korea, Kuwait, Libya, Sri Lanka, Moldova, Myanmar, Nigeria, Nicaragua, the Netherlands, Nepal, New Zealand, Oman, Panama, the Philippines, Portugal, Paraguay, Palestine, Qatar, Romania, Saudi Arabia, Sudan, Serbia, Slovakia, Sweden, Tunisia, Turkey, Uruguay, Uzbekistan, Yemen, and South Africa.

**Table 1:** Observations and CTIS^a^ respondents in mandate and nonmandate countries. Data sources: CTIS and Oxford COVID-19 Government Response Tracker.

	Countries (n=51), n (%)	Observations (n=9792), n (%)	CTIS respondents (n=8,769,994), n (%)
No mandate	8 (15.7)	1536 (15.7)	2,158,083 (24.6)
Mandate	43 (84.3)	8256 (84.3)	6,611,911 (75.4)
Total	51 (100)	9792 (100)	8,769,994 (100)

^a^CTIS: COVID-19 Trends and Impact Survey.

### Empirical Strategy

To evaluate face mask mandates, we follow the recommendations of Lison et al [23] for effectiveness assessments of nonpharmaceutical interventions. A test of our hypotheses is provided by the following potential outcomes framework: Let y_1,ct_ denote a potential outcome of interest in country c=1, 2, ..., C at calendar time t=1, 2, ..., T if a mandate is implemented. y_0,ct_ designates the potential outcome of interest in the same country if no mandate has been implemented. The average treatment effect on the outcome of interest in countries treated with mandates (average treatment effect on the treated [ATT]) is then defined as

*β* = 
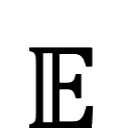
[y_1,ct_ − y_0,ct_|D_ct_=1]  **(1)**


where D_ct_=(0,1) is a mandate indicator switching on during mandate period s=1, 2, ..., S; S<T. As any given country is only observed for either D_ct_=1 or D_ct_=0, the observed outcomes of interest y_ct_ can be written


y_ct_ = y_0,ct_ + [y_1,ct_ − y_0,ct_] × D_ct_  **(2)**

When fitted to real-world data such as those generated with the CTIS, models require additional identifying assumptions to provide causal interpretations of *β*. A first assumption that is being made is that, in the absence of any mandates, outcomes would exhibit parallel trends for intervention and nonintervention countries. The parallel trends assumption ensures that the estimates of the mandate effect are not biased by unobserved time-varying heterogeneity. The identification of the average treatment effect on countries with face mask mandates, *β*, further requires that country populations do not demonstrate any self-reported anticipatory uptake of face masks or decreases in SARS-CoV-2 reproduction numbers and COVID-19 case growth rates before mandate introduction. In terms of Granger causality, a causal interpretation can be given to estimate 
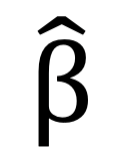
 only if the mandate effects are observed after the intervention. In addition, we assume face mask mandates to homogeneously affect all intervention countries in all periods. It must also be noted that the estimates 
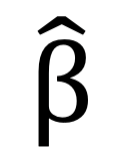
 are unbiased only under the strict exogeneity of D_ct_. Furthermore, our empirical strategy aims to estimate changes in the levels of self-reported face mask use, SARS-CoV-2 reproduction numbers, and COVID-19 case growth rates. Countries with large differences in outcome baseline levels can therefore be included in the analysis without violating the underlying assumptions regarding the functional form of the model specifications, shown below in equation 3. On the basis of the preceding assumptions, we estimate the average mandate effects on the treated countries *β* by


z_ct_ = λ_c_ + φ_t_ + 
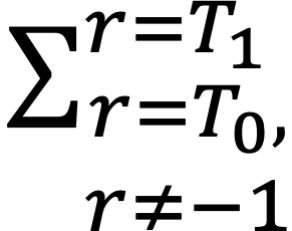
 β_t+r_D_ct+r_ + γX_ct_ + ε_ct_  **(3)**


whereby either 
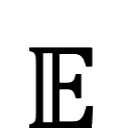
[y_ct_] = exp(z_ct_) / 1+exp(z_ct_) designates the expected share of self-reported face mask users or y_ct_=z_ct_ indicates Sars-CoV-2 reproduction numbers or case growth rates, respectively. Within this specification strategy, country and calendar time fixed effects λ_c_ and φ_t_ provide full nonparametric control for any unobserved time–constant and country-constant heterogeneity [24]. X_ct_ represents a row vector of covariates containing subgroup treatment and treatment-level indicators. Measures of COVID-19 outcomes depend on country-specific testing rates [25]. Therefore, X_ct_ also contains the number of country-specific COVID-19 tests per 100,000 inhabitants. Moreover, nonpharmacological interventions other than mask mandates as well as domestic mobility are a possible source of heterogeneity in SARS-CoV-2 reproduction numbers and COVID-19 case growth rates during the observation period. This may lead to biased estimates of the mask mandate effects. For SARS-CoV-2 reproduction numbers and COVID-19 case growth rates, X_ct_ thus includes indicators for both country-level mobility and nonpharmacological interventions other than mask mandates. γ is a column vector designating the corresponding parameters of interest. Assuming a quasi-binomial distribution of country proportions of self-reported face mask wearers, an error is given by ε_ct_ ~ Logistic(μ, ϕ), with mean μ and dispersion parameter ϕ. ε_ct_ ~ (0, σ^2^) is assumed for linear models of SARS-CoV-2 reproduction numbers and COVID-19 case growth rates. By specifying *r*=T_0_, ..., T_1_ leads and lags of the mandate effect, with T_0_<0 being the lowest and T_1_≥0 the highest number of leads and lags considered, we use a design that incorporates incremental mandate effects. This specification provides a test for the previously outlined assumption of no anticipatory events.

As countries imposed face mask mandates at different calendar dates, using already treated countries as control groups for yet-to-be treated countries is likely to induce heterogeneities of policy effects across treated units. Therefore, estimating equation 3 with conventional two-way fixed effects methods would result in biased estimates 
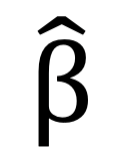
. Hence, to relax the treatment homogeneity assumption made in the preceding section, we group countries into cohorts depending on the calendar dates of mandates becoming effective, as suggested by Sun and Abraham [26]. Countries without any face mask mandates during the entire observation period are combined into a single control group. Cohort outcomes at *r*=−1, that is, the period before mandate implementation, are used as baseline outcomes. Estimates of average treatment effects are obtained by weighing the aggregated cohort-specific effects by cohort size.

### Ethical Considerations

Only publicly available data aggregated at the country level have been analyzed. This eliminates any reidentification risks for individuals. The study did not require any direct or indirect interaction with humans. This means that the regulatory requirements for research involving humans are not met. Therefore, this study is exempt from institutional review board approval.

## Results

### Descriptive Results

**Figure 1:**
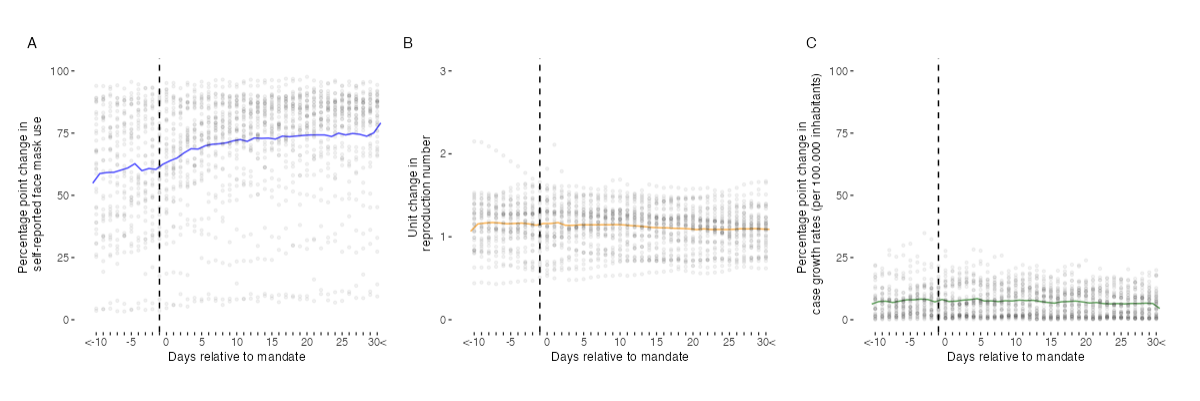
Daily average evolution of face mask use, SARS-CoV-2 reproduction numbers, and growth rates of COVID-19 cases in mandate countries. Data sources: COVID-19 Trends and Impact Survey, Oxford COVID-19 Government Response Tracker, and Our World in Data—COVID-19 data set.

Figure 1 [17,18,21] illustrates the daily average evolution of self-reported face mask use, SARS-CoV-2 reproduction numbers, and COVID-19 case growth rates in countries with mask mandates. Visual inspection of panel A reveals a substantial increase in self-reported face mask wearers around the time the regulations came into effect in these countries. As further shown in the panels B and C, no clear trends are visible for average SARS-CoV-2 reproduction numbers or growth rates of COVID-19 cases.

**Figure 2:**
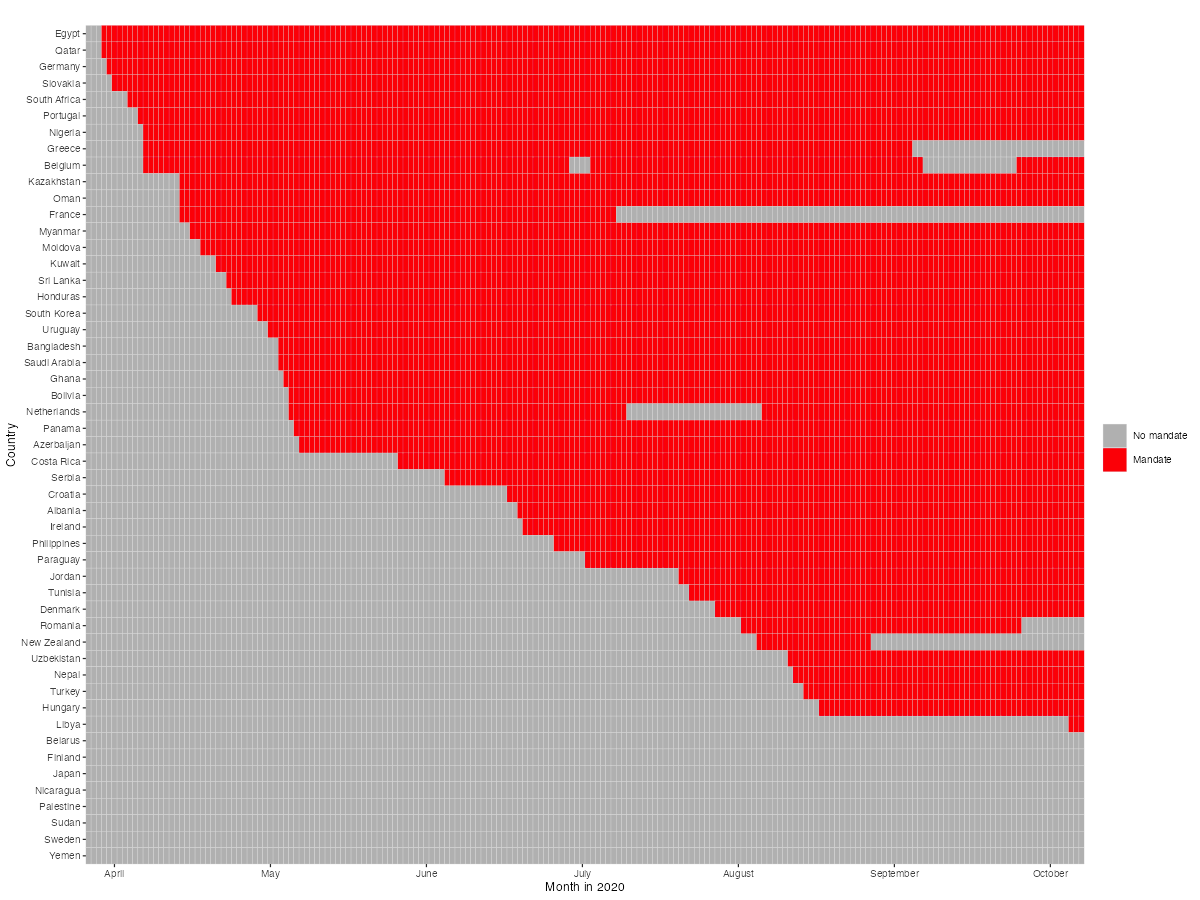
Face mask mandate periods by country. Data source: Oxford COVID-19 Government Response Tracker.

The timing of the introduction of face mask mandates in each country during the observation period is presented in Figure 2 [21]. There is a range of 186 days between the dates when mandates were introduced: although in Egypt and Qatar the wearing of masks in public was compulsory from 4 days after the start of the observation period, that is, from April 26, 2020, in Libya, it was only compulsory from October 29, 2020, that is, 3 days before the end of the observation period.

### Effects of Face Mask Mandates

We first estimated equation 3 for proportions of self-reported face mask users, SARS-CoV-2 reproduction numbers, and growth rates of COVID-19–related cases. All postmandate period effects were aggregated to the total ATT of face mask mandates. As shown in Table 2 [17,19,21,22], the results indicate that—on average—mandates are followed by an increase in self-reported face mask wearers of 8.81 percentage points (*P*=.006).

Similarly, an average decrease of −0.31 units (*P*=.008) was observed for SARS-CoV-2 reproduction numbers after face mask mandates became effective. No reduction in the growth rates of COVID-19 cases was observed after mask mandates became effective (−0.98 percentage points; *P*=.56).

**Figure 3:**
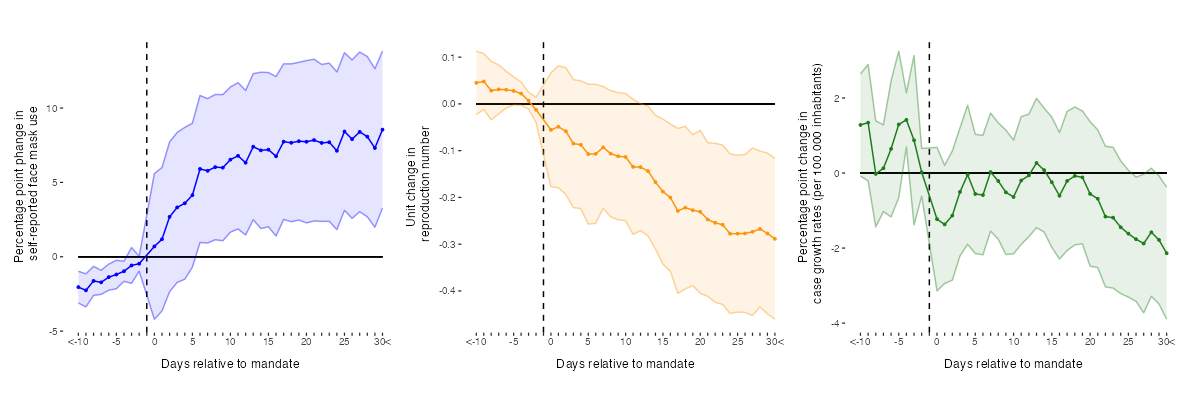
Incremental effects of face mask mandates on self-reported face mask use, SARS-CoV-2 reproduction numbers, and growth rates of COVID-19 cases. Colored points depict Sun and Abraham interaction-weighted estimates of relative period effects. Colored areas indicate 95% CI. SE are clustered at the country level. Data sources: COVID-19 Trends and Impact Survey, Oxford COVID-19 Government Response Tracker, Our World in Data—COVID-19 data set, and Google COVID-19 Community Mobility Reports.

The incremental effects of pre- and postmandate implementation periods according to the event study specification of equation 3 are presented in Figure 3 [17,19,21,22,26]. As can be seen, the incremental effects of face mask mandates are demonstrated in an increase in the proportion of self-reported mask wearers at 6 days and decreases in reproduction numbers at 13 days after mandate implementation. For self-reported face mask use and reproduction numbers, the effects continuously persist for >30 days. Furthermore, our estimations yield single mandate effects on case growth rates on days 26 (−1.76 percentage points; *P*=.04), 27 (−1.89 percentage points; *P*=.05), 29 (−1.78 percentage points; *P*=.04), and 30 (−2.14 percentage points; *P*=.02) after mandates were enacted. We observe pretrends in self-reported face mask use on up to 4 days and on day 4 in case growth rates before the beginning of the mandate periods. Our identifying assumption of no anticipatory events is supported for a period of at least 4 days before the beginning of mandates for all outcomes.

**Table 2:** Event study regressions of self-reported face mask use, SARS-CoV-2 reproduction numbers, and growth rates of COVID-19 cases on face mask mandates. Percentage point changes are shown for self-reported face mask use and growth rates of COVID-19 cases, and unit changes are shown for SARS-CoV-2 reproduction numbers. Data sources: COVID-19 Trends and Impact Survey, Oxford COVID-19 Government Response Tracker, Our World in Data—COVID-19 data set, and Google COVID-19 Community Mobility Reports.

	Self-reported face mask use^a,b^ (observations=8867)		Reproduction number^a,b^ (observations=6078)			Case growth rates^a,b^ (observations=5960)	
	Percentage points (SE)	*P* value	Units (SE)	*P* value	Percentage points (SE)		*P* value
ATT^c^	8.81 (3.08)	.006	−0.31 (0.11)	.008	−0.98 (1.66)		.56
Subgroup mandate	7.33 (4.76)	.13	0.03 (0.09)	.78	1.21 (1.54)		.44
Level-2 mandate	4.49 (4.88)	.36	0.16 (0.11)	.14	1.06 (1.17)		.37
Level-3 mandate	2.43 (4.62)	.60	0.0006 (0.11)	>.99	1.39 (2.13)		.52
School closure	—^d^	—	−0.02 (0.07)	.74	0.64 (1.01)		.53
Ban on events	—	—	−0.11 (0.05)	.05	0.23 (1.13)		.84
Ban on gatherings	—	—	−0.04 (0.04)	.32	−1.89 (1.27)		.14
Curfew	—	—	0.01 (0.06)	.93	1.49 (1.21)		.22
International travel restrictions	—	—	−0.13 (0.06)	.05	−2.43 (1.17)		.04
Protection of older adults	—	—	0.06 (0.09)	.46	−0.84 (2.89)		.77
Mobility	—	—	0.01 (0)	.10	0.51 (0.14)		<.001
Tests per 100,000 inhabitants	—	—	0 (0)	.07	0 (0)		.02

^a^All models have been estimated with country and time fixed effects.

^b^SE (in parentheses) are clustered at the country level.

^c^ATT: Average Treatment Effect on the Treated. The ATT is obtained by averaging Sun and Abraham [26] interaction-weighted estimates of postmandate period effects.

^d^The model was specified with ATT and subgroup as well as level-2 and level-3 mandate effects, only.

### Sensitivity Analyses

To assess the parsimony of the models of SARS-CoV-2 reproduction numbers and COVID-19 case growth rates, equation 3 was re-estimated by restricting the parameters of mobility and containment measures other than mask mandates to jointly equal 0. Restricted models yielded a lower mandate ATT for SARS-CoV-2 reproduction numbers (−0.32 units; *P*=.01). In line with the original specification, the results do not demonstrate any relationship between mask mandates and growth rates of COVID-19 cases (−1.22 percentage points; *P*=.43). In postmandate periods, the restricted specification also reveals no changes regarding incremental decreases in SARS-CoV-2 reproduction numbers, and our findings demonstrate a negative effect of mask mandates on reproduction numbers at 12 days after mandate beginning. The observed incremental effects last >30 days. A single incremental effect of mandates on COVID-19 case growth rates is observed on day 30 after mandate implementation (−1.84 percentage points; *P*=.03). A Wald test was used to test the null hypothesis that the parameters of mobility and containment measures other than mask mandates jointly equal 0. The null hypothesis was rejected for both models of mask mandate effects on reproduction numbers (2.67; *P*=.01) and case growth rates (6.13; *P*<.001). Therefore, we retained the initial model specification including indicators of mobility and nonpharmacological interventions other than mask mandates.

Equation 3 was also re-estimated with counterfactual linear trends to evaluate the robustness of the initial results. Compared with the baseline specification, the observed mandate ATTs are lower for self-reported face mask use (8.66 percentage points; *P*=.01) and remain unchanged for SARS-CoV-2 reproduction numbers (−0.31 units; *P*=.01). As in the original specification, no ATT is found for the growth rates of COVID-19–related cases (−0.97 percentage points; *P*=.56). The incremental increases in self-reported mask use are observed on day 6 after mandate implementation. In addition, the incremental decreases in COVID-19 reproduction numbers are demonstrated on day 12. The incremental effects persist for >30 days for both models. Both results are consistent with the original specification. Significant incremental effects of mandates on COVID-19 case growth rates are found between days 26 and 30 (−1.76 percentage points; *P*=.04 and −2.13 percentage points; *P*=.02) after the mandates take effect.

As a final sensitivity check, we have considered that the selection of mandate policies must be strictly exogenous for our specification to yield unbiased estimates of mandate effects. However, the possibility that countries enacted mask mandates depending on the course of the pandemic cannot be ruled out. To rule out bias, the mandate indicators D_ct_ were regressed on SARS-CoV-2 reproduction numbers and growth rates of COVID-19 cases, controlling for country-specific fixed effects. No significant effects were observed. In conclusion, the results of the sensitivity analyses confirm the baseline estimation results. An overview of all results from the sensitivity analyses mentioned in the preceding section is presented in Multimedia Appendix 1 [17,19,21,22,26].

### Effects of Face Mask Recommendations

The mask mandates evaluated required the populations of the countries under study to wear masks. However, in 25 (24.5%) of the 102 countries represented in the data underlying our analysis, recommendations for voluntary mask use were issued first. Therefore, the final step was to investigate whether these recommendations might have been sufficient to prevent COVID-19 transmission. Accordingly, the baseline specification of equation 3 was re-estimated, with cohorts defined depending on the calendar dates of recommendations becoming effective. Information on self-reported mask use, SARS-CoV-2 reproduction numbers, and COVID-19 case growth rates were available for 60% (15/25) of the countries at the time of face mask recommendations. A total of 11.8% (12/102) of countries were excluded from the evaluation because they demonstrated nonparallel outcome trends in the preliminary analyses. This resulted in a total of 40% (10/25) countries with face mask recommendation being included in the analyses, yielding an analysis sample incorporating 78.4% (80/102) of the countries. The control group consisted of 88% (70/80) of the countries, of which 4% (3/70) of the countries did not implement any face mask policies, whereas 96% (67/70) of the countries enacted mask mandates only.

As presented in Table 3 [17,19,21,22], no significant ATTs of mask recommendations were found for SARS-CoV-2 reproduction numbers and COVID-19 case growth rates (−0.06 units; *P*=.70 and −2.45 percentage points; *P*=.59).

However, an ATT of 5.84 percentage points (*P*<.001) was shown for mask use. In addition, increases in mask use by 11.70 percentage points (*P*=.001) and 13.60 percentage points (*P*=.002) are observed for mask mandates and subgroup mandates, respectively. Our findings also demonstrate mandate effects on reproduction numbers (−0.19 units; *P*=.04).

**Figure 4:**
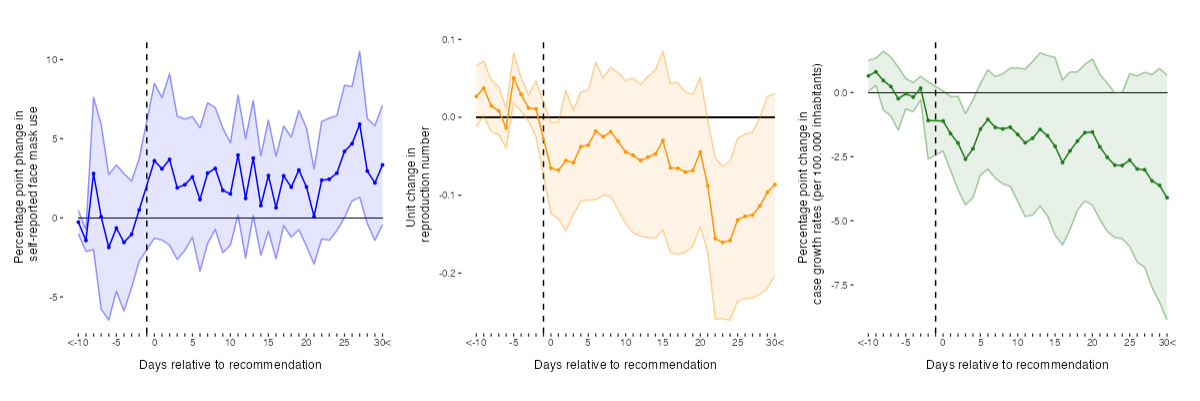
Incremental effects of face mask recommendations on self-reported face mask use, SARS-CoV-2 reproduction numbers, and growth rates of COVID-19 cases. Colored points depict Sun and Abraham interaction-weighted estimates of relative period effects. Colored areas indicate 95% CI. SE are clustered at the country level. Data sources: COVID-19 Trends and Impact Survey, Oxford COVID-19 Government Response Tracker, Our World in Data—COVID-19 data set, and Google COVID-19 Community Mobility Reports.

Figure 4 [17,19,21,22,26] presents the effects of face mask recommendations in 10 countries. As can be inferred, recommendations had an isolated incremental effect on self-reported use of face masks on days 11 (3.96 percentage points; *P*=.04), 13 (3.77 percentage points; *P*=.04), and 25 to 27 (4.20 percentage points; *P*=.048 and 5.91 percentage points; *P*=.01) after publication. Single incremental effects of mask recommendations are also observed for reproduction numbers on days 0 (−0.07 units; *P*=.03) and 1 (−0.07 units; *P*=.03) and between days 21 (−0.09 units; *P*=.04) and 28 (−0.11 units; *P*=.05) after publication. Case growth rates decrease incrementally between days 1 and 4 (−1.60 percentage points; *P*=.03 and −2.19 percentage points; *P*=.03) and on day 23 (−2.83 percentage points; *P*=.05) after recommendations were published. Pretrends are shown for >10 days and on day 9 before the recommendation for self-reported face mask use, on days 9, 5, and 4 for SARS-CoV-2 reproduction numbers, and on days 10 and 9 for growth rates of COVID-19–related cases. These findings support the assumption of no anticipatory events for at least 3 days before face mask recommendations.

**Table 3:** Event study regressions of self-reported face mask use, SARS-CoV-2 reproduction numbers, and growth rates of COVID-19 cases on face mask recommendations. The table reports percentage point changes in self-reported face mask use and growth rates of COVID-19 cases as well as unit changes in reproduction numbers. Data sources: COVID-19 Trends and Impact Survey, Oxford COVID-19 Government Response Tracker, Our World in Data—COVID-19 data set, and Google COVID-19 Community Mobility Reports.

	Self-reported face mask use^a,b^ (observations=13,159)			Reproduction number^a,b^ (observations=8500)			Case growth in cases^a.b^ (observations=8325)	
	Percentage points (SE)	*P* value	Units (SE)		*P* value	Percentage points (SE)		*P* value
ATT^c^	5.84 (1.60)	<.001	−0.06 (0.16)		.70	−2.45 (4.54)		.59
Mask mandate	11.70 (3.44)	.001	−0.19 (0.09)		.04	−2.01 (1.36)		.15
Subgroup mandate	13.60 (4.34)	.002	−0.18 (0.12)		.16	−3.18 (2.22)		.16
Level 2—mandate	1.16 (3.20)	.72	0.05 (0.08)		.54	2.22 (1.38)		.11
Level 3—mandate	−1.27 (3.88)	.74	−0.08 (0.10)		.42	0.76 (1.99)		.70
School closure	—^d^	—	0.07 (0.05)		.12	1.13 (0.92)		.22
Ban on events	—	—	−0.12 (0.05)		.02	−0.38 (0.77)		.62
Ban on gatherings	—	—	−0.02 (0.05)		.70	−0.70 (1.02)		.50
Curfew	—	—	−0.09 (0.04)		.02	−0.81 (0.66)		.23
International travel restrictions	—	—	−0.09 (0.06)		.15	−1.58 (1.41)		.27
Protection of older adults	—	—	0.04 (0.05)		.42	−0.78 (1.50)		.61
Mobility	—	—	−0.01 (0.01)		.26	0.37 (0.098)		<.001
Tests per 100,000 inhabitants	—	—	0 (0)		.20	0 (0)		.02

^a^All models have been estimated with country and time fixed effects.

^b^SE (in parentheses) are clustered at the country level.

^c^ATT: Average Treatment Effect on the Treated. The ATT is obtained by averaging Sun and Abraham [26] interaction-weighted estimates of postmandate period effects.

^d^The model was specified with ATT and subgroup as well as level-2 and level-3 mandate effects, only.

## Discussion

### Principal Findings

According to the results presented in the preceding section, the introduction of face mask mandates was associated with an increase in self-reported face mask use and a decrease in SARS-CoV-2 reproduction numbers. The sustainability of face mask mandates is demonstrated by the fact that the observed effects persist for >30 days after the mandates come into effect. Our results are robust to different sensitivity analyses. Thus, during our observation period, face mask mandates proved to be an effective nonpharmacological measure to reduce SARS-CoV-2 reproduction numbers.

The effectiveness of mask use has occasionally been questioned in public debate [9]. This is particularly true in light of the inconclusive findings of Jefferson et al [5]. According to the authors, a key problem with the existing RCTs is that they have a wide variation in outcome measures, making it difficult to compare results. Using real-world data, our study allows a comparison of SARS-CoV-2 reproduction numbers and COVID-19 case growth rates across 51 countries. This increases the external validity of previous findings, which are largely based on RCTs. Furthermore, our results support the findings of Ueki et al [27] and Cheng et al [28]. On the basis of aerosol and droplet transmission models, both papers show that face masks are effective in reducing the risk of transmission in low virus load environments such as public spaces.

In addition, our observations suggest inconclusive evidence on the effect of mask mandates on the growth rates of COVID-19 cases. Although Chernozhukov et al [29] demonstrate that mask mandates have contributed to a reduction in case growth rates at the US state level, we found no differences in COVID-19 cases between mandate and nonmandate countries. The lack of mask mandate effects in our study may be, at least in part, owing to the high infectiousness of the SARS-CoV-2 virus. With such high infectiousness, the implementation of mask mandates may not be sufficient to prevent clusters of COVID-19 cases in the most susceptible populations, such as older adults or those who are immunosuppressed. This may be particularly true for facilities such as older people’s care homes, where most cases occurred. Inadequate compliance with mask mandates or improper use of face masks by infected individuals in contact with these populations is hardly always avoidable. Therefore, the occurrence of such clusters is difficult to prevent. Additional strict containment measures may have been required to protect the most susceptible populations.

For masks to have a preventive effect, they must be worn by the public. Accordingly, Jefferson et al [5] discuss the lack of mask adherence as a possible cause of the inconclusive results of RCTs on the effectiveness of face masks in preventing ARIs. To increase the willingness to wear masks, public health authorities used social media to educate the public during the COVID-19 pandemic [30] or distributed masks free of charge [31]. In addition to these interventions, governments have introduced more drastic initiatives, such as mandatory masks, which penalize people who do not wear masks. Adding to the existing evidence from RCTs, our study shows that the self-reported use of masks is significantly increased because of such regulations. Although this is consistent with a previous study that found high compliance with mandatory mask use [12], our results further indicate that mandates, rather than recommendations, are required to ensure self-reported mask adherence.

### Limitations

It cannot be completely ruled out that the estimates of self-reported face mask use are biased because of the sampling method used. First, the data on self-reported face mask use are obtained from country-specific samples of Facebook users. The distribution of these samples may not reflect the composition of each country’s population. The survey weights calculated by Meta, which are based on population- and region-specific gender and age distributions, may only partially compensate for such biases. Another possible limitation is that it can be assumed that the course of infection is determined by individual behavior. No microlevel data were available for this study that would have allowed us to test this assumption and accurately estimate behavioral effects on the effectiveness of mask mandates. A third complication is that the available data do not allow to distinguish between the type of mask worn (surgical mask vs N95 mask). Depending on mask type, the effects of mandates on reproduction numbers may differ. Such differences cannot be estimated within the framework of this study.

### Conclusions

Our results suggest that mask mandates encourage self-reported mask use and reduce SARS-CoV-2 reproduction numbers. They may be a simple measure in a nonpharmacological strategy to control epidemics caused by respiratory-transmissible pathogens. When implementing mask mandates, special care should be taken to ensure compliance with respect to the most susceptible individuals. Given the lack of mandate effects on the growth rates of COVID-19 cases, additional containment measures may be required to ensure the adequate protection of the most susceptible populations.

## Appendix

Multimedia Appendix 1 Additional results from sensitivity analyses.

## Abbreviations

ARI: acute respiratory infection
ATT: average treatment effect on the treated
CTIS: COVID-19 Trends and Impact Survey
OxCGRT: Oxford COVID-19 Government Response Tracker
RCT: randomized controlled trial

## Data Availability

The data sets generated during and analyzed during this study are available from the corresponding author on reasonable request.
