# Supplementary material for: Effects of Face Mask Mandates on COVID-19 Transmission in 51 Countries: Retrospective Event Study
Source: JMIR Public Health Surveill. 2024 Mar 8;10:e49307. doi: 10.2196/49307 (PMC10926949; doi:10.2196/49307)
Supplement: Multimedia Appendix 1 [file publichealth_v10i1e49307_app1.docx]

**Appendix**

**A1 – Restricted estimation excluding indicators of mobility and non-pharmacological interventions other than mask mandates**

**Figure S1** – Incremental effects of face mask mandates on SARS-CoV-2 reproduction numbers and growth rates of COVID-19 cases. Estimations exclude indicators of mobility and non-pharmacological interventions other than mask mandates. Colored points depict Sun and Abraham interaction-weighted estimates of relative period effects [26]. S.E. are clustered at the country level. Data sources: COVID-19 Trends and Impact Survey [17], Oxford COVID-19 Government Response Tracker [21], Our World in Data – COVID-19 Dataset [19] & Google COVID-19 Community Mobility Reports [22].


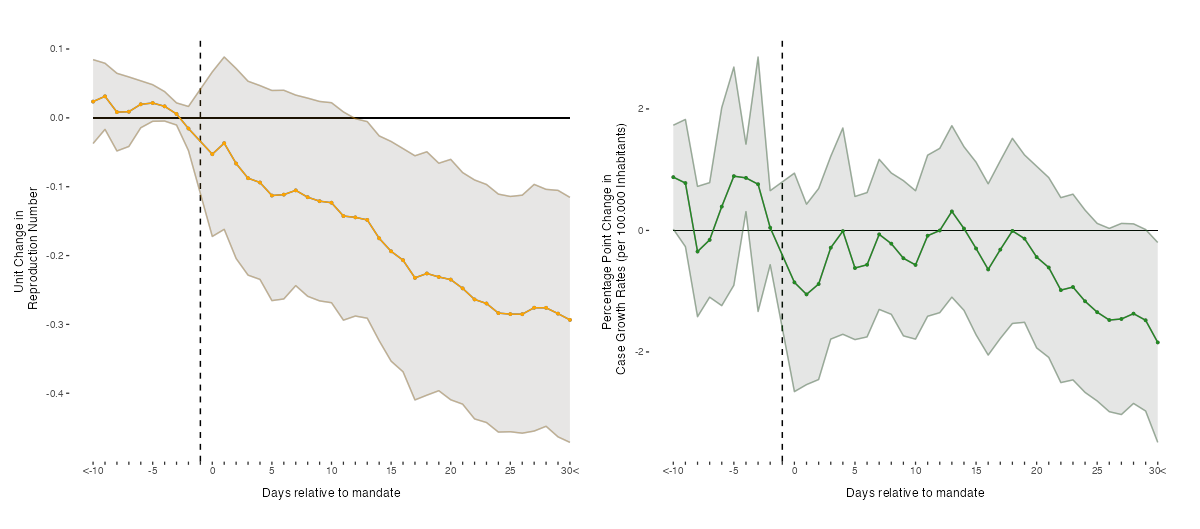


**Table S1 –** Wald test for the null hypothesis that parameters of mobility and containment measures other than mask mandates estimated in the initial specification (see Table 2 in the main text) jointly equal zero. S.E. are clustered at the country level. Data sources: COVID-19 Trends and Impact Survey [17 ], Oxford COVID-19 Government Response Tracker [21], Our World in Data – COVID-19 Dataset [19] & Google COVID-19 Community Mobility Reports [22].

|  | Reproduction Number | Growth in Cases |
| --- | --- | --- |
| Wald-Test | 2.67  P=.01 | 6.13  P<.001 |
| Observations | 6,270 | 6,137 |

**Table S2 –** Event-study-regressions of SARS-CoV-2 reproduction numbers and growth rates of COVID-19 cases on face mask mandates. Estimations exclude indicators of mobility and non-pharmacological interventions other than mask mandates. The table reports percent point changes of self-reported face mask use and growth rates of COVID-19 cases and unit changes of SARS-CoV-2 reproduction numbers. S.E. (in parentheses) are clustered at the country level. The ATT is obtained by averaging Sun and Abraham interaction-weighted estimates of post-mandate period effects [26]. ‘Inh.’ and ‘FE’ are abbreviations from ‘Inhabitants’ and ‘Fixed Effects’, respectively. Data sources: COVID-19 Trends and Impact Survey [17], Oxford COVID-19 Government Response Tracker [21], Our World in Data – COVID-19 Dataset [19] & Google COVID-19 Community Mobility Reports [22].

|  | Reproduction Number | Growth in Cases |
| --- | --- | --- |
| ATT | -0.32 (0.11)  P=.01 | -1.22 (1.51)  P=.43 |
| Subgroup Mandate | 0.04 (0.09)  P=.63 | 0.21 (1.68)  P=.90 |
| Level 2 - Mandate | 0.12 (0.10)  P=.26 | -0.39 (1.44)  P=.79 |
| Level 3 - Mandate | 0.02 (0.10)  P=.86 | 1.56 (2.07)  P=.46 |
| Tests per 100k Inh. | 0.0 (0.00)  P=.07 | 0.0 (0.00)  P=.04 |
| Country-FE | Yes | Yes |
| Time-FE | Yes | Yes |
| Observations | 6,270 | 6,137 |

**A2 – Estimation with counterfactual linear trends**

Counterfactual linear trends $\hat{y}$_ct_ = $\hat{z}$_ct_ were calculated by extrapolating pre-policy trends to post-mandate periods as according to:

| $\hat{y}$_ct_ = $\hat{z}$_ct_ = $\hat{\tau}$_P_t + λ_c_, | (A1) |
| --- | --- |

where P indicates pre-mandate periods for mandate countries and all periods for non-mandate countries. $\hat{\tau}$ represents the coefficient estimate for calendar time t and λ_c_ designates a country fixed effect. For sensitivity analysis, the predictions $\hat{y}$_ct_ = $\hat{z}$_ct_ in country c were included as covariates in the estimation of equation (3).

**Figure S2** – Incremental effects of face mask mandates on self-reported face mask usage, SARS-CoV-2 reproduction numbers and growth rates of COVID-19 cases. Estimations include counterfactual linear trends. Colored points depict Sun and Abraham interaction-weighted estimates of relative period effects [26]. S.E. are clustered at the country level. Data sources: COVID-19 Trends and Impact Survey [17], Oxford COVID-19 Government Response Tracker [21], Our World in Data – COVID-19 Dataset [19] & Google COVID-19 Community Mobility Reports [22].


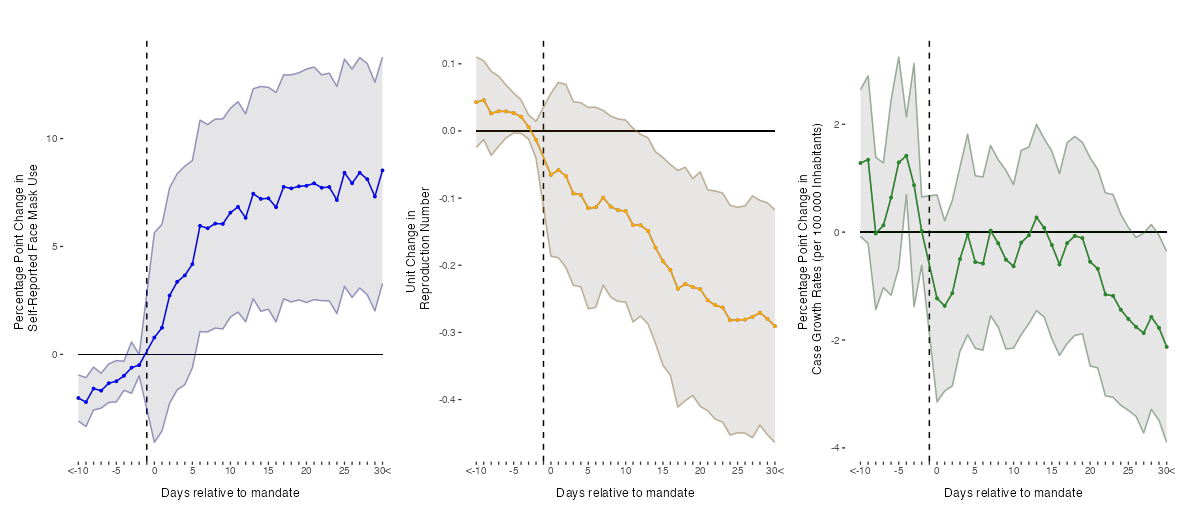


**Table S3** – Event-study-regressions of face mask usage, SARS-CoV-2 reproduction numbers and growth rates of COVID-19 cases on face mask mandates. Estimations include counterfactual linear trends. The table reports percent point changes of face mask use and growth rates of COVID-19 cases as well as unit changes of SARS-CoV-2 reproduction numbers. S.E. (in parentheses) are clustered at the country level. The ATT is obtained by averaging Sun and Abraham interaction-weighted estimates of post-mandate periods [26]. ‘Inh.’ and ‘FE’ are abbreviations from ‘Inhabitants’ and ‘Fixed Effects’, respectively. Data sources: COVID-19 Trends and Impact Survey [17], Oxford COVID-19 Government Response Tracker [21], Our World in Data – COVID-19 Dataset [19] & Google COVID-19 Community Mobility Reports [22].

|  | Face Mask Usage | Reproduction Number | Growth in Cases |
| --- | --- | --- | --- |
| ATT | 8.66 (3.10)  P=.01 | -0.31 (0.12)  P=.01 | -0.97 (1.66)  P=.56 |
| Subgroup Mandate | 7.17 (4.71)  P=.13 | 0.02 (0.10)  P=.82 | 1.21 (1.53)  P=.43 |
| Level 2 - Mandate | 4.14 (4.87)  P=.40 | 0.18 (0.10)  P=.097 | 1.05 (1.19)  P=.38 |
| Level 3 - Mandate | 2.21 (4.58)  P=.63 | 0.03 (0.11)  P=.81 | 1.38 (2.15)  P=.52 |
| School Closure | - | -0.03 (0.07)  P=.64 | 0.629 (1.01)  P=.54 |
| Ban on Events | - | -0.11 (0.06)  P=.06 | 0.224 (1.12)  P=.84 |
| Ban on Gatherings | - | -0.04 (0.05)  P=.43 | -1.89 (1.27)  P=.14 |
| Curfew | - | 0.01 (0.06)  P=.93 | 1.50 (1.21)  P=.22 |
| International Travel Restrictions | - | -0.12 (0.06)  P=.05 | -2.43 (1.17)  P=.04 |
| Protection of the Elderly | - | 0.06 (0.09)  P=.50 | -0.84 (2.89)  P=.77 |
| Mobility | - | 0.01 (0.00)  P=.11 | 0.513 (0.141)  P<.001 |
| Tests per 100k Inh. | - | 0.00 (0.00)  P=.08 | 0.00 (0.00)  P=.01 |
| Country-FE | Yes | Yes | Yes |
| Time-FE | Yes | Yes | Yes |
| Counterfactual Linear Trends | Yes | Yes | Yes |
| Observations | 8,796 | 6,063 | 5,947 |

**A3 – Exogeneity check of face mask mandates**

In order to test whether the introduction of mandates was influenced by country-specific COVID-19 trajectories or any time-varying unobservables, the following logistic regression was estimated:

| Pr(D_ct_ = 1) = exp($\delta$y_ct_ + λ_c_) / 1 + exp($\delta$y_ct_ + λ_c_) | (A3) |
| --- | --- |

Within this specification, coefficient estimates $\hat{\delta}$ capture effects of COVID-19 outcomes y_ct_ on the likelihood of mandate adoption Pr(D_ct_ = 1) or any unobserved time-varying heterogeneity. y_ct_ designates either current Sars-CoV-2 reproduction number or weekly growth rates of COVID-19 cases and deaths.

**Table S4 –** Logistic panel regressions of face mask mandates, SARS-CoV-2 reproduction numbers and growth rates of COVID-19 cases. The table reports percent point changes of face mask use and growth rates of COVID-19 cases and unit changes of SARS-CoV-2 reproduction numbers. S.E. (in parentheses) are clustered at the country level. ‘FE’ is abbreviated from ‘Fixed Effects’. Data sources: Oxford COVID-19 Government Response Tracker [21] & Our World in Data – COVID-19 Dataset [19].

|  | Face Mask Mandate | Face Mask Mandate |
| --- | --- | --- |
| Reproduction Number | 0.05 (0.58)  P=.93 | – |
| Case Growth Rate | – | -0.05 (0.04)  P=.37 |
| Country-FE | Yes | Yes |
| Observations | 7,692 | 7,224 |
